# Cell-free DNA and circulating *TERT* promoter mutation for disease monitoring in newly-diagnosed glioblastoma

**DOI:** 10.1186/s40478-020-01057-7

**Published:** 2020-11-04

**Authors:** Maxime Fontanilles, Florent Marguet, Ludivine Beaussire, Nicolas Magne, Louis-Ferdinand Pépin, Cristina Alexandru, Isabelle Tennevet, Chantal Hanzen, Olivier Langlois, Fabrice Jardin, Annie Laquerrière, Nasrin Sarafan-Vasseur, Fréderic Di Fiore, Florian Clatot

**Affiliations:** 1 Inserm U1245, Normandie Univ, UNIROUEN, IRON group, Normandy Centre for Genomic and Personalized Medicine, Rouen, France; 2 Department of Medical Oncology, Cancer Centre Henri Becquerel, Rue d’Amiens, 76000 Rouen, France; 3 Normandie Univ, UNIROUEN, Inserm U1245, Normandy Centre for Genomic and Personalized Medicine, Rouen, France; 4 Department of Pathology, Rouen University Hospital, 76031 Rouen, France; 5 Department of Radiology, Rouen University Hospital, 76031 Rouen, France; 6 Department of Statistics and Clinical Research Unit, Henri Becquerel Cancer Center, Rouen, France; 7 Department of Radiation Oncology and Medical Physics, Cancer Centre Henri Becquerel, Rue d’Amiens, 76000 Rouen, France; 8 Department of Neurosurgery, Rouen University Hospital, 76031 Rouen, France; 9 Department of Gastroenterology, Rouen University Hospital, 76031 Rouen, France; 10 Département d’oncologie médicale, Centre de Lutte Contre le Cancer Henri Becquerel, Rue d’Amiens, 76038 Rouen, France

**Keywords:** Glioblastoma, Cell-free DNA, Circulating tumor DNA, *TERT* promoter mutation, Liquid biopsy

## Abstract

The clinical implications of plasmatic cell-free and tumor DNA (cfDNA and ctDNA) are challenging in glioblastoma. This prospective study included 52 consecutive newly diagnosed glioblastoma (n = 49) or gliosarcoma (n = 3) patients treated with concomitant temozolomide and radiotherapy (RT-TMZ), followed by a TMZ maintenance phase. Plasma samples were collected at baseline, before RT-TMZ (pre-RT-TMZ) and at the end of adjuvant TMZ, or at the time of progression in cases of progressive disease (PD). The cfDNA concentration was measured with a fluorometric method, and ctDNA was detected using targeted droplet digital PCR. The main objectives were to analyze the associations between cfDNA and ctDNA measurements during the course of treatment with PD and survival. There was a significant decrease in median cfDNA concentration from baseline to pre-RT-TMZ—19.4 versus 9.7 ng/mL (*p* < 0.0001)—in the entire cohort. In patients with PD, a significant increase in cfDNA concentration from pre-RT-TMZ to time of PD was observed, from 9.7 versus 13.1 ng/mL (*p* = 0.037), respectively, while no difference was observed for nonprogressive patients. Neither the cfDNA concentration at baseline nor its kinetics correlated with survival. ctDNA was detected in 2 patients (3.8%) and only in gliosarcoma subtypes.

*Trial registration* ClinicalTrial, NCT02617745. Registered 1 December 2015, https://clinicaltrials.gov/ct2/show/NCT02617745?term=glioplak&draw=2&rank=1.

**Electronic supplementary material:**

The online version of this article (10.1186/s40478-020-01057-7) contains supplementary material, which is available to authorized users.

## Introduction

Glioblastoma is the most frequent primary adult brain tumor, with 125,000 to 150,000 new cases per year worldwide [1]. First-line treatment for glioblastoma is based on surgical resection whenever possible, followed by radiotherapy (RT) plus concomitant and adjuvant temozolomide (TMZ) [2]. The prognosis remains poor, with a median overall survival time of less than 20 months. Radiological evaluation using magnetic resonance imaging (MRI), including perfusion and diffusion-weighted sequences, is considered to be the most appropriate method for both tumor burden evaluation and disease monitoring. The diagnosis of recurrence is often challenging because pseudo progression may also occur. A reliable definition of patients with progressive and nonprogressive disease is required both for clinical decision-making and for evaluating responses within clinical trials.

In this context, the use of circulating cell-free DNA (cfDNA) is considered very promising to optimize the management of these patients. cfDNA is related to cell turnover, combining both circulating normal DNA and tumor DNA (ctDNA). The cfDNA concentration is reportedly associated with prognosis in many cancers, while ctDNA, which harbors somatic alterations, was shown to be a surrogate marker for disease monitoring [3, 4]. As yet, the clinical relevance of these markers is not clearly established in patients with glioblastoma because, in addition to technological considerations, the presence of the blood–brain barrier physically limits the crossing of small genetic fragments [5]. The literature is conflicting regarding the role of cfDNA and ctDNA in glioblastoma. Indeed, although these markers are frequently reported to be informative in cerebrospinal fluid (CSF), their detection rate in plasma has been variable in several studies, ranging from 7.9 to 55% [6, 7]. Glioblastoma harbors multiple recurrent somatic alterations within *epithelial growth factor receptor* (*EGFR*), *phosphatase and tensin homolog* (*PTEN), TP53* or *telomerase reverse transcriptase* (*TERT*) genes [8]. *TERT* encodes a subunit of the telomerase complex. Somatic mutations within the *TERT* promoter (*TERTp*) region are involved in oncogenesis in various type of tumor including glioblastoma [9]. Two recurrent mutations of *TERTp* that reach 70–80% frequency in glioblastoma [10, 11] are located − 124 bp and − 146 bp upstream of the *TERT* translation start site (chromosome 5p15.33: 1,295,228 C > T and 1,295,250 C > T, respectively C228T and C250T). Thus, *TERTp* mutations may be a potential biomarker for diagnosis [12] and disease monitoring in glioblastoma.

In this context, the aim of this prospective study was to evaluate the cfDNA concentration and the detection rate of ctDNA at diagnosis and during the course of treatment, as well as their correlation with tumor progression, in patients treated for a newly diagnosed glioblastoma.

## Materials and methods

### Study population, tumor and plasma collection

Patients with newly diagnosed, histologically confirmed, supratentorial glioblastoma or gliosarcoma, according to the World Health Organization classification 2016 [13], were prospectively included in our study from July 2016 to November 2017. Patients had to be at least 18 years of age and planned to receive RT (60 Gy in 30 fractions) with concomitant TMZ therapy, followed by adjuvant TMZ with or without previous radical surgery [2]. Patients with concomitant or previous history of cancer within 2 years were excluded. Blood samples were prospectively collected according to the following schedule: at baseline before resection or before biopsy in cases of nonresected tumor; before the initiation of RT-TMZ (pre-RT-TMZ); and at the end of adjuvant TMZ treatment, corresponding to the 6th or the 12th cycles according to the physician’s choice. In cases of progressive disease (PD), a blood sample was collected at the time of progression, before the end of adjuvant TMZ. All patients were followed according to the RANO criteria [14] using brain MRI every 3 months from the end of the RT-TMZ phase.

All MRIs performed at baseline were reviewed for tumor volume determination. They were analyzed on a T2-Flair and T1 postcontrast sequences after manual segmentation using Advantage Windows 4.6^®^ (General Electric Healthcare, Milwaukee, Wisconsin, United States). The analysis of tumor volume was performed in T1 postcontrast sequences according necrotic areas. The maximal relative cerebral blood volume (rCBV) was also measured by perfusion-weighted imaging, when available, by drawing several regions of interest in high-rCBV areas in the tumor and was compared to the contralateral non-affected brain parenchyma [15, 16].

Samples of 6 mL of whole blood were collected at three different time points into tubes containing ethylenediaminetetraacetic acid (EDTA) based on previous studies [17, 18]. Within 2 h after blood collection, the tubes were centrifuged at 3000 rpm for 10 min; the plasma was then extracted and stored at − 80 °C until use. Tumor samples were obtained during diagnostic procedures (resection or biopsy). After collection, the tumor samples were used for routine histopathology, immunohistochemistry and molecular biology analyses (Additional file 1). Another tumor sample was flash-frozen at − 80 °C and stored until use. Ten healthy subjects were included as plasma sample controls. The study was an ancillary of the ongoing prospective GLIOPLAK trial, registered in ClinicalTrials.gov (NCT02617745, https://clinicaltrials.gov/ct2/show/NCT02617745). Informed written consent to participate in the study was obtained from all patients, and the French National Committee for the Protection of Persons approved the study (RCB ID 2015-A00377-42).

### Cell-free DNA extraction and quantification

cfDNA was extracted from 1 to 5 mL of plasma using the QIAamp Circulating Nucleic Acid kit^®^ (Qiagen, Hilden, Germany) according to the manufacturer’s instructions. The sample was eluted in a final volume of 30 µL and stored at − 20 °C. Double-stranded DNA quantification was performed by a fluorometric method using a Quantit™ PicoGreen^®^ dsDNA Assay kit (Invitrogen, Carlsbad, CA, USA) and a Twinkle LB970 microplate fluorimeter (Berthold, Bad Wildbad, Germany). For each sample, cfDNA quantification was performed in duplicate from 2 µL of eluate, and normalization was performed using a standard calibration curve of known concentrations of standard dsDNA (from 0 to 10 ng).

The fragment distribution of the cfDNA was analyzed using the Agilent 4200 TapeStation^®^ System (Santa Clara, CA USA) with D5000 High-Sensitivity ScreenTape. This system uses electrophoresis to separate DNA fragments from 100 to 5000 base pairs (bp). The mean DNA length is then automatically estimated for each peak.

### Circulating tumor DNA exploration by droplet digital PCR

ctDNA detection was analyzed according to *telomerase reverse transcriptase* promoter (*TERTp)* mutation detection by droplet digital PCR (ddPCR) (Additional file 1). The limit of detection (LOD) was determined from the plasma of healthy subjects according to a previously reported method [12, 13]. Healthy subjects were volunteers free from acute or chronic pathology; plasma samples were collected and stored under the same pre-analytical conditions as for the patients. Short-sized *TERTp* sequencing was performed using a ddPCR-based method with an amplicon of 88 bp (reference dHsaEXD20945488 for *TERTp* C228T and reference dHsaEXD85215261 for *TERTp* C250T). The experimental procedure was identical to that used for the 113 bp assays. Positive-case control was a patient suffering from *TERTp*-mutated hepatocellular carcinoma.

Matched tumor DNA was extracted from formalin-fixed paraffin-embedded tissue and sequenced using ddPCR 113 bp assay following the previously-described procedure.

### Statistical analyses

The objective was to evaluate variations of cfDNA and ctDNA and their associations with baseline characteristics and outcome. The analysis was performed in the whole population and according to the presence (or not) of a resected tumor. The outcome was analyzed according to the rate of PD at 6 and 12 months, as well as progression-free survival (PFS) and overall survival (OS). OS was defined as the time from diagnosis to the date of death or last follow-up. PFS was defined as the time from diagnosis to the date of first disease progression or date of death. Prognostic factors such as the Karnofsky performance status index, type of surgical procedure (biopsy versus resection) and *O6*-*methylguanine*-*DNA methyltransferase* promoter (*MGMTp*) methylation status were explored in univariate and multivariate analyses. OS and PFS were calculated using Kaplan–Meier method and compared with the log–rank test. Multivariate analyses were performed using variables in univariate analyses with a *P* value less than or equal to 10%. Follow-up period for survival analyzes was set at 12 months; in case of non-progressive disease monitoring continued up to 2 years (median 4 months). The data were compared using Student’s test for normally distributed continuous variables or the Mann–Whitney–Wilcoxon test for nonparametric variables; the Fisher exact test or Pearson’s Chi squared test was used for categorical variables, as appropriate. A difference was considered statistically significant if the degree of significance (*p* value) was less than or equal to 0.05 (alpha risk = 5%). Statistical analyses were performed using the R software (R version 3.5.1, 2018, Vienna, Austria) [19].

## Results

### Patients’ characteristics

A total of 52 patients were included. As shown in Table 1, 62% (32/52) underwent surgical resection of the primary tumor, and all patients underwent an RT-TMZ sequence. Based on a median follow-up of 12 months (2–25 months), 41 patients (78%) had PD and 28 died (54%). Among patients with PD, 30 patients (58%) had progression during the TMZ maintenance phase. The median PFS was 8.5 months [95% confidence interval 6–13], and the median OS was 17 months [95% CI 16–21]. Blood samples were available for 90% of the patients (47/52) at baseline, for 100% of the patients (52/52) before RT-TMZ initiation, and for 90% of the patients (47/52) for the third sample, 5 patients died before the third blood collection. The third sample was collected from 47 patients either at the 6th (n = 10) or at 12th TMZ cycle (n = 12) or at the time of PD (n = 25).

**Table 1:** Clinical and tumor characteristics

Characteristics	Number of patients (%)
Age (years), mean ± SD	58.3 ± 9.5
Sex	
Female	22 (42.3%)
Male	30 (57.7%)
Surgery	
Biopsy	20 (38.4%)
Resection	32 (61.6%)
Karnofsky performance score	
≥ 80%	45 (86.5%)
≥ 70 to < 80%	6 (11.5%)
< 70%	1 (2%)
*Tumor Characteristics*	
Volume at baseline, mean ± SD	
T1-weighted with Gadolinium W/necrosis	31.1 cm^3^ ± 28.6
T1-weighted with Gadolinium W/O necrosis	18 cm^3^ ± 16.5
Necrosis part, mean (%)	33% [min. 0–max. 87%]
Flair	114.9 cm^3^ ± 63.2
Histology	
Glioblastoma-*IDH* wild type	46 (88.4%)
Glioblastoma *IDH*-mutated	3 (5.8%)
Gliosarcoma	3 (5.8%)
*MGMT*p methylation	
Non-methylated	26 (50%)
Low methylation profile	2 (4%)
Methylated	12 (23%)
Unknown	12 (23%)
*TERTp* mutation	
C228T	44 (84.6%)
C250T	1 (1.9%)
Wild-type (wt)	6 (11.6%)
Unknown	1 (1.9%)

### Baseline cfDNA

**Fig. 1:**
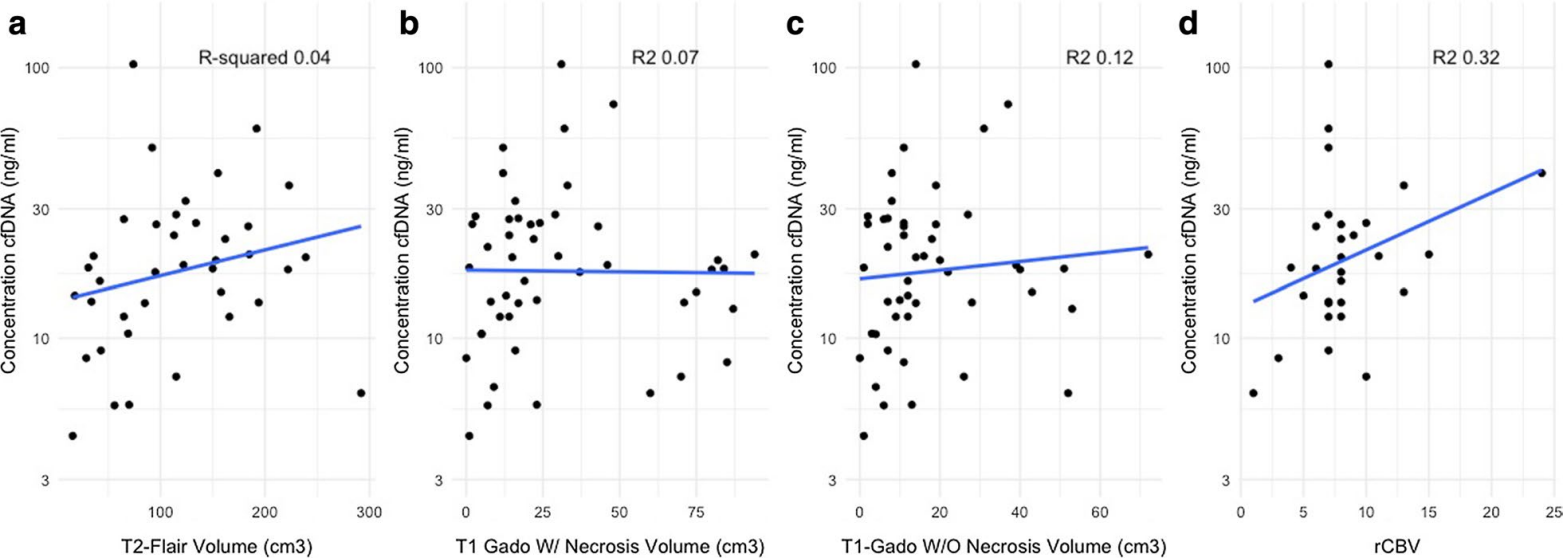
Correlations between cell-free DNA concentration at baseline and MRI characteristics. The cfDNA concentration is plotted against three tumor volumes: **a** T2-Flair; **b** T1-enhanced volume with necrosis; **c** T1 enhanced volume without necrosis (*p* = 0.018); and **d** rCBV (*p* = 0.03). rCBV had the highest association with cfDNA concentration. Gado: gadolinium; W/: with; W/O: without; rCBV: relative cerebral blood volume

The median cfDNA concentration was 19.4 ng/mL (interquartile range (IQR) 13.6–27.6) compared to 5.6 ng/mL (IQR 5.1–6.6) in the group of 10 healthy volunteers, *p* < 0.0001. The cfDNA concentration has a significant although weakly positive association with the T1-weighted gadolinium-enhanced volume without necrosis (R^2^ 0.12, *p* = 0.018) but not with the FLAIR volume or the T1-weighted gadolinium-enhanced volume with necrosis (Fig. 1c, a, b respectively). The rCBV was measured for 27 patients (mean = 9.03 [range 4–24]) and correlated with the cfDNA concentration (R-squared 0.32, *p* = 0.03) (Fig. 1d). The cfDNA tended to be higher in patients treated with corticosteroid (n = 30) compared to patients who were not (n = 17) (21 ng/mL versus 14.4 ng/mL (*p* = 0.1), respectively). The level of cfDNA was not correlated with CRP (R^2^ 0.001, *p* = 0.71) or albuminemia (R^2^ 0.005, *p* = 0.5).

### Correlations between cfDNA variations during treatment and clinical outcome

A significant decrease in median cfDNA concentration was observed from baseline to pre-RT-TMZ, 19.4 ng/mL versus 9.7 ng/mL (*p* < 0.0001) respectively, corresponding to a mean decrease of − 53% (range − 94 to − 1%). The cfDNA decrease was observed both in the biopsy group and in the resection group (16.9 ng/mL vs. 10.5 ng/mL (*p* = 0.022) and 20.4 ng/mL vs 9.4 ng/mL, (*p* < 0.001), respectively). Decrease in corticosteroid exposure was observed between baseline and pre-RT-TMZ; and could explain the cfDNA decrease in the entire cohort: mean equivalent prednisolone dose of 63 mg at baseline versus 32 mg at pre-RT-TMZ (*p* = 0.01). Thus, a non-significant decrease in corticosteroid dose in the biopsy group (54 mg at baseline vs. 42 mg at pre-RT-TMZ, *p* = 0.43) could partly explain the decrease of cfDNA in the biopsy group.

**Fig. 2:**
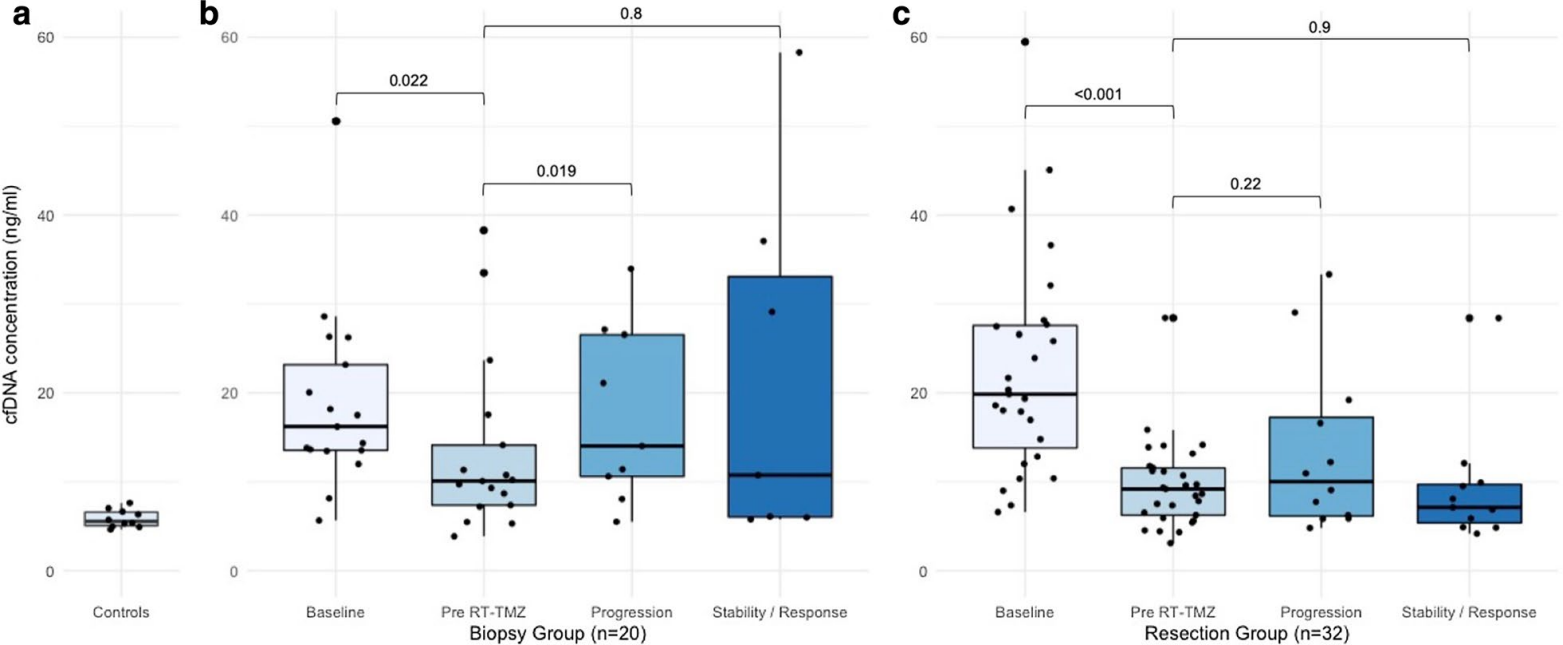
Variations of circulating cell-free DNA concentration during treatment. **a** Median cfDNA concentration (ng/mL of plasma) at baseline is higher than in the control group (healthy subjects) and decreases between baseline and pre-RT-TMZ in both the biopsy group (**b**) and the tumor resection group (**c**). RT: radiotherapy; TMZ: temozolomide

Among the 22 patients without PD during the adjuvant TMZ phase, no difference in the cfDNA concentrations was observed between pre-TMZ and the end of the adjuvant TMZ phase (9.7 vs. 9.5 ng/mL (*p* = 0.6), respectively). In contrast, among the 30 patients who experienced PD during the TMZ phase, we observed a significant increase in the median cfDNA level from pre-TMZ to the time of progression (9.7 vs. 13.1 ng/mL (*p* = 0.037), respectively) (Additional file 2: Table S1). The increase in cfDNA was mainly observed among patients with biopsy only, 10.5 ng/mL versus 21.1 ng/mL (*p* = 0.019) (Fig. 2), and was independent of corticosteroid dose adjustment, with a mean equivalent prednisolone dose of 42 mg at pre-RT-TMZ versus 51 mg at progression (*p* = 0.73).

**Fig. 3:**
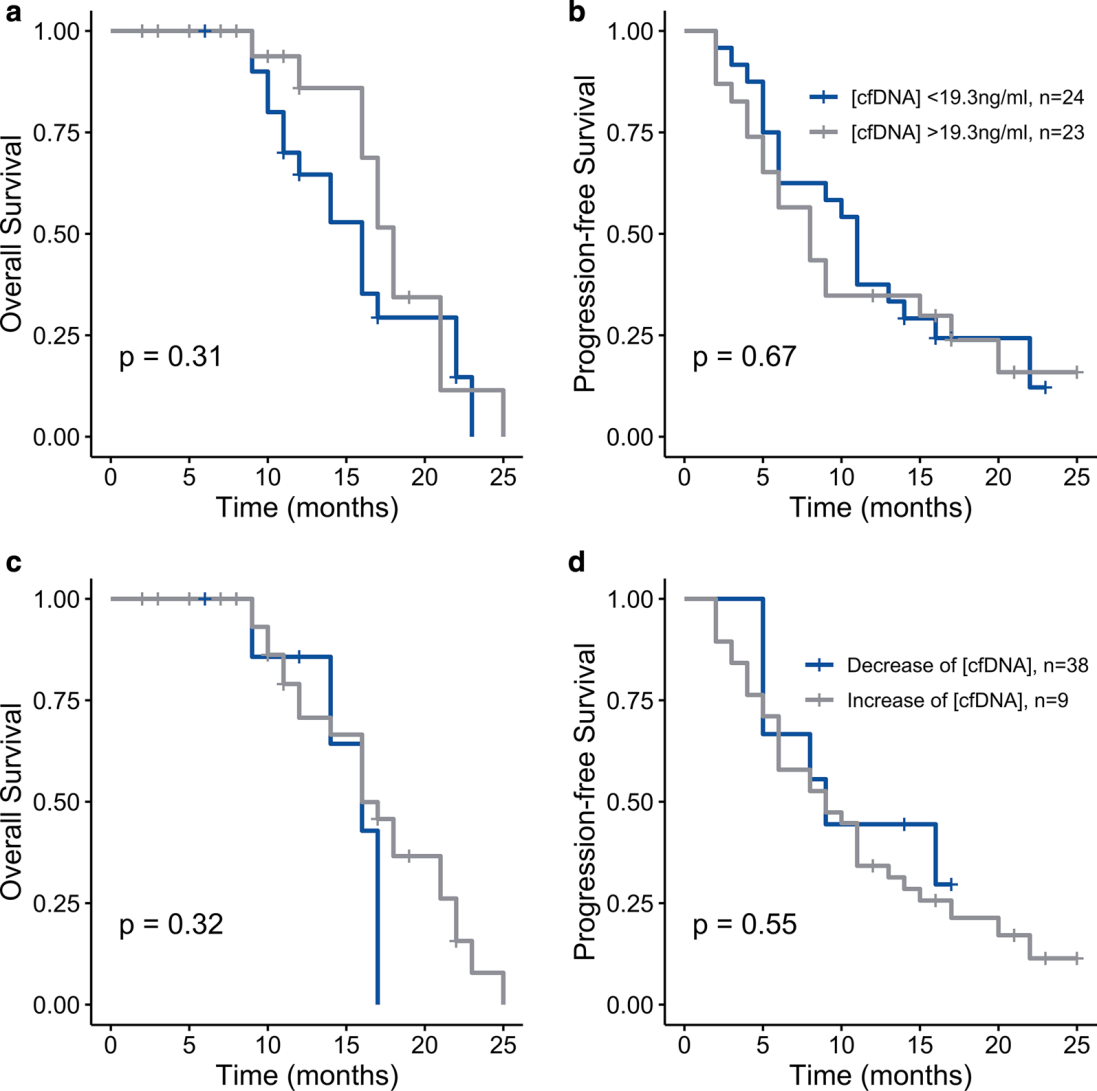
Survival curves. **a** Overall survival and progression-free survival **b** according to baseline cfDNA concentration below or above the median. OS (**c**) and PFS (**d**) according to the increase or decrease of cfDNA between baseline and pre-RT-TMZ

In the entire cohort, the baseline cfDNA concentration and its variation before the RT-TMZ phase were not associated with OS or PFS (Fig. 3, Additional file 3: Table S2). In the subgroup of patients with resection, the baseline cfDNA concentration and its kinetics before the RT-TMZ phase did not influence overall survival or progression-free survival (data not shown).

The cfDNA size estimation by the TapeStation^®^ method was performed for 51/52 patients for at least one of the three time point samples. Overall, the median DNA size was 86 bp [IQR 67.5–596], with 63% of the DNA fragments less than 113 bp. No difference in DNA size according to the timing of samples was observed (Additional file 2: Table S1). There was no association between DNA size and cfDNA concentration.

### ctDNA detection using *TERTp* mutation

**Fig. 4:**
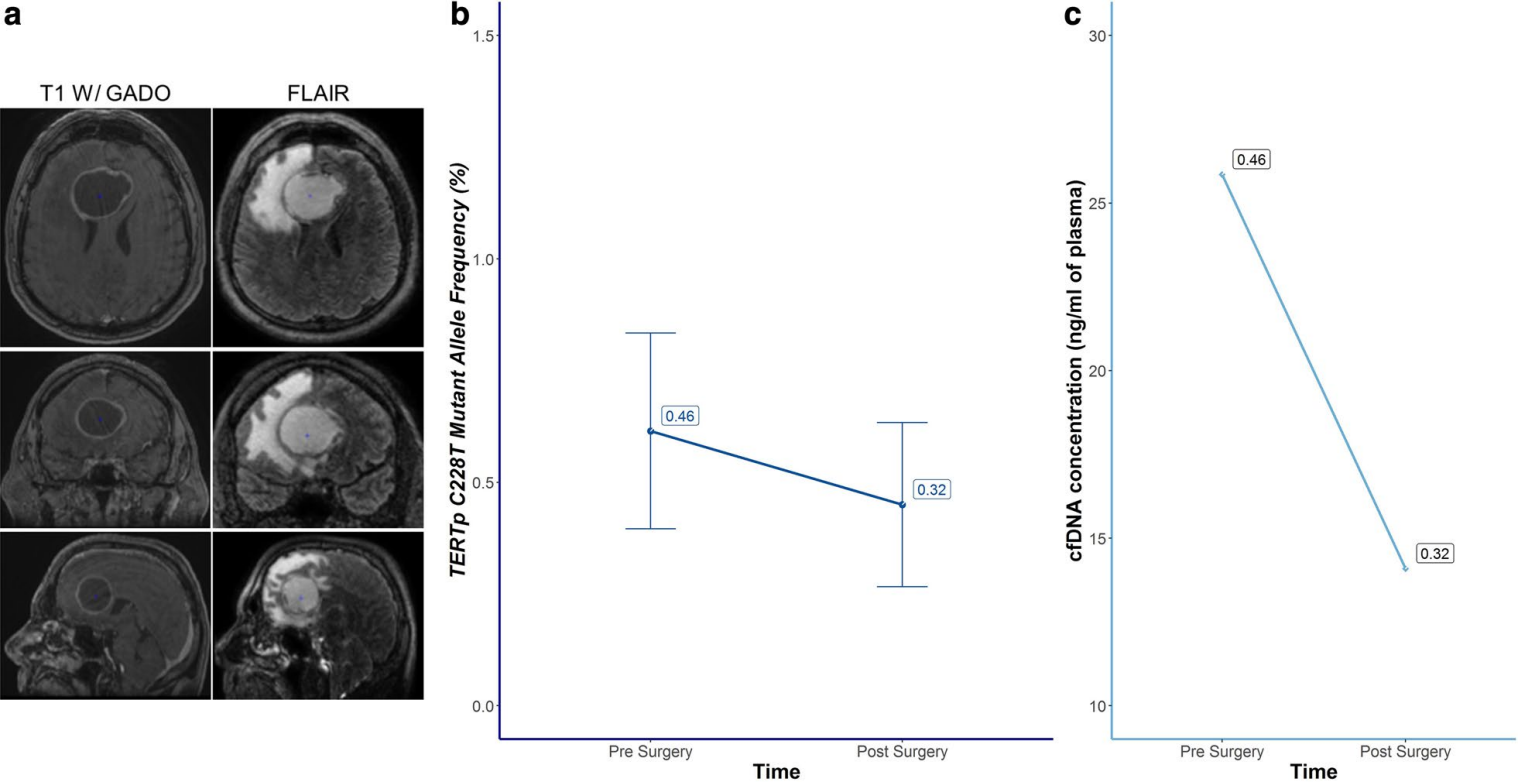
Patient (#059) with *TERTp* C228T gliosarcoma and *TERTp* detection in plasma at diagnosis. Patient #059 was a 64-year-old man suffering from frontal C228T *TERTp*, *IDH* wild-type gliosarcoma spreading into the corpus callosum. **a** The lesion was predominantly necrotic: 43 cm^3^ of T1-weighted gadolinium volume with necrosis versus 11 cm^3^ without necrosis; the percentage of necrosis was 74%. In contrast, the rest of the cohort had an average rate of necrosis of 33%. The T2-FLAIR volume was also consequential, with a total volume of 184 cm^3^. **b** Circulating C228T *TERTp* mutation was detected both pre- and postoperatively. Two assays were performed (assay 1 and 2). The presented results are the mean of the two assays, with error bar (± standard deviation). **c** The kinetics of cfDNA concentration correlated with ctDNA MAF and decreased after tumor resection

**Fig. 5:**
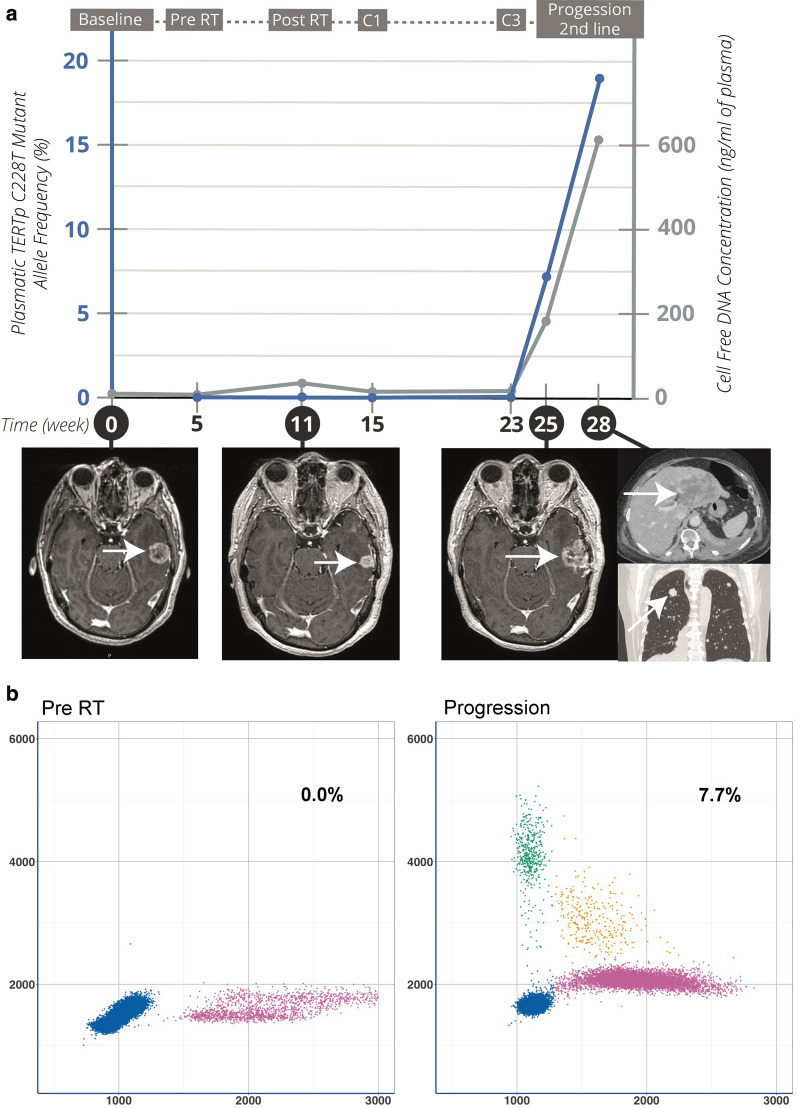
Plasma longitudinal follow-up of the *TERTp* C228T mutation in a patient (#082) with *TERTp*-mutated gliosarcoma. **a** Patient #082 was a 69-year-old woman suffering from left temporal unmethylated *MGMTp*, *IDH* wild-type (wt) gliosarcoma with C228T *TERTp* mutation (MAF 28.6% in initial resected tumor using ddPCR). Partial tumor resection was initially performed. The plot shows the evolution of MAF and plasma cfDNA concentration as a function of time. C228T *TERTp* mutation was not detected in plasma until cerebral relapse, with sphenoid bone invasion. The longitudinal plasma samples taken during the first-line treatment schedule revealed a correlation between circulating C228T *TERTp* MAF, cfDNA concentration, and the evolution towards metastatic status. **b** Two representations of the search for *TERTp* C228T mutation in plasma at baseline (left) and at progression (right). Channel 1 fluorescence (vertical axis, FAM™) is plotted against channel 2 (horizontal axis, HEX™). Droplets are grouped as clusters: FAM™/HEX™negative (double-negative droplets, blue points), FAM™positive/HEX™negative (green points), FAM™negative/HEX™positive (pink points), and FAM™/HEX™positive (double-positive droplets, orange points)

Plasma samples from 46 patients (46/52, 88.4%) were available for ctDNA *TERTp* C228T and C250T 113 bp ddPCR assays. Among them, 2 (2/46, 4.3%) had a detectable circulating *TERTp* mutation in at least one sample. The first patient had a circulating *TERTp* C228T mutation detected at baseline and at pre-RT-TMZ (Fig. 4). The second patient had *TERTp* C228T detected only at time of PD, when distant metastases were diagnosed (Fig. 5). Interestingly, *TERTp* detection in plasma seemed to be restricted to the gliosarcoma subtype: 2/3 (67%) versus 0/49 for glioblastoma. *TERTp* mutation analysis using the ddPCR 88 bp assay did not increase the detection rate. Interestingly, 2 plasma samples (3.8%) harbored at least 2 droplets using a duplicate 88 bp assay and were considered as negative according to the positive threshold (Additional file 4: Table S3, Additional file 5: Figure S1).

## Discussion

Our results, based on serial blood samples collected prospectively, suggest that the evaluation of cfDNA concentration, in contrast to *TERTp* mutation from plasmatic ctDNA, may give clinically relevant information in patients treated with RT-TMZ for glioblastoma. At baseline, we observed that the cfDNA level was associated with tumor neoangiogenesis based on MRI parameters (R-squared 0.32 regarding association between rCBV and cfDNA concentration). Our results also highlighted that cfDNA underwent significant variation during the treatment course, with a significant decrease after initial surgery or biopsy procedures and a significant increase in cases of PD during the TMZ maintenance phase. As yet, the role of cfDNA in patients treated for glioblastoma has not been clearly established. In a subgroup of 12 patients, Bagley et al. recently reported that cfDNA increased in cases of PD and remained stable in nonprogressive patients, similar to our findings [7]. Moreover, the interest of cfDNA as a reflection of tumor burden was previously reported in a series showing that its level may correlate with radiological parameters. Taken together, these findings support the hypothesis that cfDNA is released from the vascularized part of the tumor [20] and that this marker might be a useful tool for disease monitoring in the TMZ maintenance phase.

The evaluation of plasmatic ctDNA has been rarely investigated in prospective studies of patients with glioblastoma treated with RT-TMZ. In our work, only 2 cases with detectable circulating *TERTp* mutation (n = 2/52, 3.8%) were observed using ddPCR, whereas 85% of the tumors were mutated, which was in accordance with previous studies [11, 21]. The *TERTp* mutation detection rate was similar to that of Juratli et al., showing a rate at baseline of 7.9% by nested PCR in 38 patients [6]. ddPCR was used for *TERTp* mutation detection, because this sequencing method is an ultrasensitive, fast and cost-effective tool, enabling its routine use in clinical practice. The lack of detected *TERTp* mutations in plasma could be related to the short size of the ctDNA fragments (< 70 bp), which are not able to be detected by current sequencing methods [22]. ddPCR is a sensitive method for detecting DNA fragments with amplicon sizes of 113 bp and 88 bp for *TERTp* C228T and *TERTp* C250T. Unfortunately, the use of the 88 bp amplicon size did not increase the sensitivity of ddPCR in our study. These results are to be balanced by the fact that some samples presented very few mutated droplets (samples considered as negative regarding our LOD) and that the quantity of DNA used for the 88 bp assay was less than that used for the 113 bp assay. Another point of discussion to increase detection rate would be to increase the supply of ctDNA. In our study, the patients had an average of 6 mL of whole blood per collection time, which appeared ethically acceptable. The question of increasing the volume of blood collected to increase the detection of ctDNA could be asked in a dedicated methodological study. Although the detection of circulating *TERTp* mutation is a rare, our results also underlined that its detection may be of particular interest in two situations: extraneural metastatic dissemination, as previously reported in a case of metastatic ependymoma [12], and the gliosarcoma subtype. Extraneural metastatic dissemination of gliomas is a rare, end-stage event, occurring in less than 2% of all cases [23] and might be linked to blood–brain barrier rupture with hematogenous dissemination favored by first-line surgery [24]. Unlike glioblastoma, gliosarcoma is characterized by its ability to invade the skull and disseminate systemically [13]. It has been postulated that in cases of gliosarcoma, the blood–brain barrier has increased permeability, with a consequently greater release of ctDNA into the general blood circulation [25]. In contrast to the ddPCR method, the use of targeted next-generation sequencing (NGS) would likely increase the ctDNA detection rate by identifying other recurrent altered genes [7], but this did not correspond to our primary objective which was to identify a single biomarker of disease evolution. The clinical utility, as well as the sensitivity, of ctDNA detection in plasma by ddPCR and the NGS method require further characterization in larger cohorts, especially compared to CSF [26, 27]. The choice of a non-selected population was made in order to explore the interest of the liquid biopsy concept in the daily-practice of patients with grade IV glioma (resection and biopsy, glioblastoma *IDHwt*, glioblastoma *IDHmut*, gliosarcoma). Regarding our results, a prospective study dedicated to the gliosarcoma or bulky *IDHwt* glioblastoma subgroup would be of major interest.

Finally, the survival analyzes failed to show an impact of cfDNA, in contrast to the survival data reported by Bagley et al. [7]. The survival results should be interpreted with caution due to the lack of power. Prospective and dedicated data are warranted to identify any prognostic significance of the cfDNA baseline concentration. An early cfDNA decrease after a diagnostic procedure should be investigated in a larger homogeneous cohort by separately analyzing resected tumor patients and bulky tumor patients. In addition, it would be interesting to explore the impact on survival of cfDNA kinetics before and right after RT-TMZ phase, before TMZ maintenance phase. The specific impact of corticosteroid therapy on the tumor release of cfDNA remains unclear despite a trend of association observed in our cohort at baseline (21 ng/mL vs. 14.4 ng/mL (*p* = 0.1)). There is a high likelihood that the prednisolone equivalent dose and the tumor volume are confounding factors for cfDNA. During TMZ treatment, the cfDNA concentration continues to vary according to the tumor evolution—independent of the corticosteroid dose adjustment. Conversely, very high doses of corticosteroid delivered as a bolus could contribute to cfDNA release into the bloodstream and may explain the early cfDNA decrease between biopsy and the pre-RT-TMZ time point in this specific subgroup for whom no oncologic treatment was administered. As previously reported in other cancers, a transient spike of cfDNA may be observed right after tumor procedure [28]. In our cohort we did not investigate this early cfDNA variation due to the lack of blood sample few days after diagnostic procedure. This early variation should be interesting to analyze regarding glioblastoma and biopsy procedure in further studies. No association between cfDNA and CRP, as inflammatory state marker, was observed. However, it would be interesting in further studies to analyze the relationship between cfDNA, corticosteroid, plasmatic inflammatory markers and white blood cell counts.

## Conclusion

Our results showed that, in contrast to ctDNA using *TERTp* mutation detection, the cfDNA concentration varies significantly over the course of treatment and may be a biomarker of PD during the TMZ phase.

## Additional files

**Additional file 1**. Supplementary methods.

**Additional file 2: Table S1**. Comparison of cfDNA characteristics at different sampling times.

**Additional file 3: Table S2**. Results of the Cox model for overall survival and progression-free survival.

**Additional file 4: Table S3**. Raw data from *TERTp* C228T and C250T sequencing with 113bp and 88bp assays per patient in the entire cohort.

**Additional file 5: Figure S1.** Analysis of the cfDNA fragment sizes and impact on ctDNA detection. **a** Control case: A patient suffering from *TERTp*-mutated hepatocellular carcinoma. Highly sensitive (HS) TapeStation^®^ electrophoresis identified a DNA peak at 132 bp (left). Total cfDNA sequencing confirmed the presence of ctDNA by detecting the circulating *TERTp* mutation, with an allelic mutation frequency of 19.6% (right). **b** Patient with *TERTp* glioblastoma. HS TapeStation^®^ electrophoresis identified a DNA peak at 80 bp. 113 bp *TERTp* ddPCR assay did not detect any positive droplet; **c** whereas 88 bp *TERTp* ddPCR assay identified positive droplets in duplicate experiments. This case remained negative for circulating *TERTp* detection regarding the positive threshold due to the very low MAF

## Data Availability

Deidentified data are available in supplementary material.

## Abbreviations

bp: base pair
cfDNA: cell-free DNA
ctDNA: circulating cell-free tumor DNA
CI: confidence interval
CSF: cerebrospinal fluid
ddPCR: digital droplet polymerase chain reaction
EDTA: ethylenediaminetetraacetic acid
*IDH*: *isocitrate dehydrogenase*
IQR: interquartile range
LOD: limit of detection
MAF: mutant allele frequency
MGMT: O-6-methylguanine-DNA methyltransferase
MRI: magnetic resonance imaging
NGS: next generation sequencing
PFS: progression free survival
PD: progressive disease
OR: odds ratio
OS: overall survival
RANO: response assessment in neuro-oncology
rCBV: relative cerebral blood volume
RT: radiotherapy
*TERT*: *telomerase reverse transcriptase*
TMZ: temozolomide
