# Supplementary material for: Cell-free DNA and circulating TERT promoter mutation for disease monitoring in newly-diagnosed glioblastoma
Source: Acta Neuropathol Commun. 2020 Nov 4;8:179. doi: 10.1186/s40478-020-01057-7 (PMC7641818; doi:10.1186/s40478-020-01057-7)
Supplement: Supplementary file 1 — Additional file 1. Supplementary methods. [file 40478_2020_1057_MOESM1_ESM.docx]

**SUPPLEMENTAL METHOD**

The histological diagnosis of glioblastoma or gliosarcoma was performed on slides stained by haemalun-eosin in agreement with WHO 2016 criteria (cytonuclear atypia, mitoses, necrosis, endothelio-capillary proliferation, associated sarcomatous component). The diagnosis was confirmed by immunohistochemistry using the following antibodies: GFAP (RBK037-05 clone EP672Y rabbit, dilution 1/200, Diagomics®, France), OLIGO2 (AC-0106RUOB clone EP112 rabbit, dilution 1/200, Clinisciences®, France) and index of proliferation KI67 (M724001 clone MIB-1 mouse, dilution 1/100, Dako®, Agilent®, California, UNITED STATES). To better characterize glial tumors at molecular level, the following antibodies were also used: IDH1^R132H^ (DIA H09 clone H09 mouse, dilution 1/50, Dianova®, Canada), ATRX (HPA001906 rabbit, dilution 1/200, Sigma Aldrich®, Missouri, UNITED STATES) and H3K27me3 (C15410195 rabbit, dilution 1/300, Diagenode®, New Jersey, United States). To highlight the associated sarcomatous component, vimentine antibody was used (M072501 clone V9 mouse, dilution 1/1000, Dako®).

Tumor DNA were extracted from formalin-fixed and paraffin-embedded using the Maxwell 16 FFPE Plus LEV DNA Purification® kit on Maxwell 16 Instrument® (Promega®, Fitchburg®, Wisconsin, United States). *IDH1* c.394C, *IDH1* c.395G, *IDH2* c.514A, *IDH2* c.515G, *IDH2* c.516G mutations on exon 4 were analyzed using ABI PRIM SNaPshot® Multiplex Kit on 3130XL Genetic Analyzer. Limit of detection was set at 5% of mutant allele frequency for at least 10% of tumor cells. *MGMTp* methylation was analyzed with pyrosequencing method (Therascreen MGMT Pyro®, Qiagen®) after DNA bisulfite reaction with EZ DNA methylation-gold kit® (Zymo research®, Proteigene®). Mean of methylated CpG islands lower than 8%: non-methylated; 9-12%: low methylation profile; greater than 12%: methylated *TERTp.*

ddPCR experiments were performed using a Qx200® ddPCR System (Biorad®, Hercules, CA, USA) using expert design assays to detect C228T and C250T *TERTp* mutations (reference dHsaEXD72405942 and dHsaEXD46675715 respectively). Those assays have an amplicon size of 113bp. ddPCR were run in a final volume of 20µL containing 10µL ddPCR Supermix for Probes (no dUTP), 2µL Betaine 5M, 0.25µL EDTA 80Mm, 0.25µL CviQI enzyme (10U/µl), 1µL 20X of *TERT* Assay and 6.5µL of extracted cfDNA. Thermal cycling was performed according to the manufacturer’s instructions: 10min at 95°C; then 50 cycles at 96°C for 30s, 62°C for 1min; and a final step of 10min at 98°C. The software Quantasoft® was used for the interpretation of the profiles.
