# Supplementary figures and images for: Cell-free DNA and circulating TERT promoter mutation for disease monitoring in newly-diagnosed glioblastoma
Source: Acta Neuropathol Commun. 2020 Nov 4;8:179. doi: 10.1186/s40478-020-01057-7 (PMC7641818; doi:10.1186/s40478-020-01057-7)

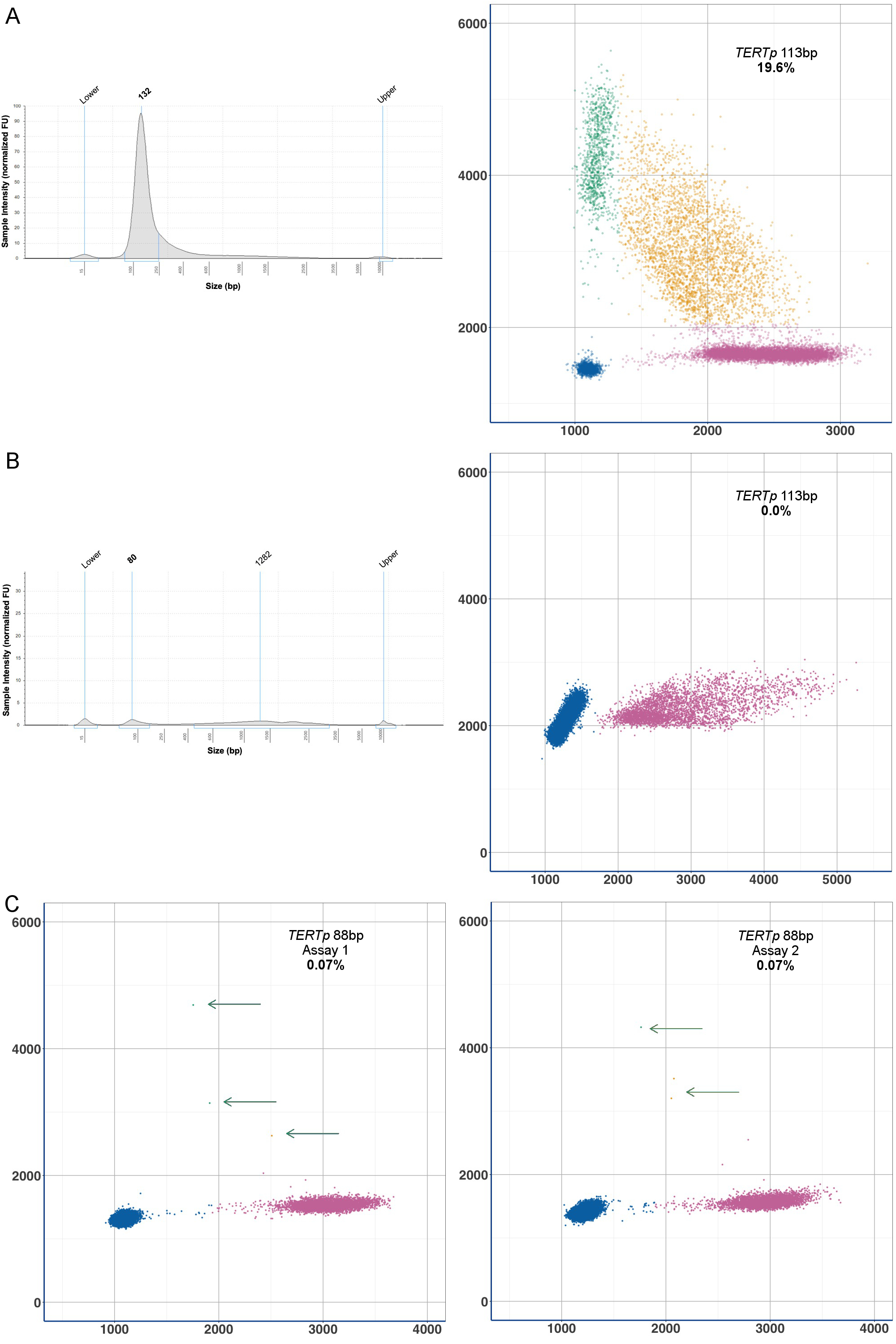

Supplement: Supplementary file 5 — Additional file 5: Figure S1. Analysis of the cfDNA fragment sizes and impact on ctDNA detection. a Control case: A patient suffering from TERTp-mutated hepatocellular carcinoma. Highly sensitive (HS) TapeStation® electrophoresis identified a DNA peak at 132 bp (left). Total cfDNA sequencing confirmed the presence of ctDNA by detecting the circulating TERTp mutation, with an allelic mutation frequency of 19.6% (right). b Patient with TERTp glioblastoma. HS TapeStation® electrophoresis identified a DNA peak at 80 bp. 113 bp TERTp ddPCR assay did not detect any positive droplet; c whereas 88 bp TERTp ddPCR assay identified positive droplets in duplicate experiments. This case remained negative for circulating TERTp detection regarding the positive threshold due to the very low MAF [file 40478_2020_1057_MOESM5_ESM.jpeg]
